# Hepatitis B Core-Related Antigen and New Therapies for Hepatitis B

**DOI:** 10.3390/microorganisms9102083

**Published:** 2021-10-02

**Authors:** Takehisa Watanabe, Takako Inoue, Yasuhito Tanaka

**Affiliations:** 1 Department of Gastroenterology and Hepatology, Faculty of Life Sciences, Kumamoto University, Kumamoto 860-8556, Japan; twatanabe@kumadai.jp; 2 Department of Clinical Laboratory Medicine, Nagoya City University Hospital, Nagoya 467-8602, Japan; clinoue@med.nagoya-cu.ac.jp

**Keywords:** hepatitis B core-related antigen, HBcrAg, iTACT-HBcrAg, chronic hepatitis B, silent cccDNA

## Abstract

The hepatitis B core-related antigen (HBcrAg) is an unprecedented novel HBV biomarker that plays an essential role in reflecting covalently closed circular DNA (cccDNA) in chronic hepatitis B (CHB) because its levels correlate with intrahepatic cccDNA and serum HBV DNA. In this review, we describe the clinical application of serum HBcrAg in CHB patients, with a particular focus on new therapies targeting intrahepatic HBV replication. (1) HBcrAg can be detected in clinical cases where serum HBV DNA is undetectable during anti-HBV therapy. (2) A highly sensitive HBcrAg assay (iTACT-HBcrAg) may be useful for monitoring HBV reactivation, as an alternative to HBV DNA. (3) Decreased HBcrAg levels have been significantly associated with promising outcomes in CHB patients, reducing the risk of progression or recurrence of hepatocellular carcinoma. Additionally, we focus on and discuss several drugs in development that target HBV replication, and monitoring HBcrAg may be useful for determining the therapeutic efficacies of such novel drugs. In conclusion, HBcrAg, especially when measured by the recently developed iTACT-HBcrAg assay, may be the most appropriate surrogate marker, over other HBV biomarkers, to predict disease progression and treatment response in CHB patients.

## 1. Introduction

Hepatitis B, caused by the hepatitis B virus (HBV), is a lethal viral infection which can lead to acute and chronic hepatitis. Chronic hepatitis B (CHB) can lead to liver diseases such as cirrhosis and hepatocellular carcinoma (HCC) [1]. CHB affects about 300 million people universally (World Health Organization; https://www.who.int/news-room/fact-sheets/detail/hepatitis-b (accessed on 20 September 2021) and it is estimated that 15–40% of them will develop cirrhosis and/or HCC [2]. In spite of the introduction of an effective hepatitis B vaccine, CHB remains a vital health problem globally and carries a high risk of mortality [3]. Most patients with CHB have a promising clinical course; however, HBV infection eventually leads to cirrhosis, liver failure, or HCC in a large number of patients [4].

Serological biomarkers of HBV are very important for predicting the course of CHB and reflect intrahepatic HBV replication activity as noninvasive alternatives to liver biopsy. On the other hand, the covalently closed circular DNA (cccDNA) present in the nuclei of infected hepatocytes cannot be eliminated, so it is important to determine its amount and activity. However, an invasive test is required to examine cccDNA directly.

Hepatitis B core-related antigen (HBcrAg) is an unprecedented, new HBV marker that plays a pivotal role in reflecting cccDNA in CHB because it is associated with not only serum HBV DNA but also intrahepatic cccDNA. Additionally, it is significantly associated with better outcomes in CHB patients with undetectable serum HBV DNA and HBsAg when HBcrAg levels are low or undetectable. HBcrAg can predict hepatitis B e antigen (HBeAg) seroconversion to antibody (anti-HBe) positivity during the course of the disease, persistent pre- and post-stop responses to nucleoside analogues, probable HBV reactivation, and risk of HCC development or recurrence. In addition, a highly sensitive HBcrAg (iTACT-HBcrAg) assay may be useful for monitoring reactivation as a very sensitive HBV cccDNA activation marker and as a substitute for HBV DNA testing.

This review describes the role of serum HBcrAg testing in the treatment of CHB, with a particular focus on new therapies targeting intrahepatic HBV replication. In addition, monitoring of HBcrAg may help determine therapeutic efficacy, as many new prospective therapeutic anti-HBV agents are premised on concomitant use of nucleoside analogues (NAs).

## 2. The Natural History of HBV Infection

The prospect that infection of an individual with HBV will become persistent depends on their age at infection [5]. Almost 90% of infants infected with HBV and 25–50% of children infected between 1 and 5 years old develop chronic hepatitis B. Over 25% of them will later develop cirrhosis and HCC [6]. The frequency of development of cirrhosis and HCC is <1% per year for patients in the immunologically inactive phase of chronic hepatitis, although the rate of development of cirrhosis may be 2–10% per year for patients in the active phase. The progression rate from cirrhosis to HCC in adult patients may be in 2–4% per year [7].

**Figure 1:**
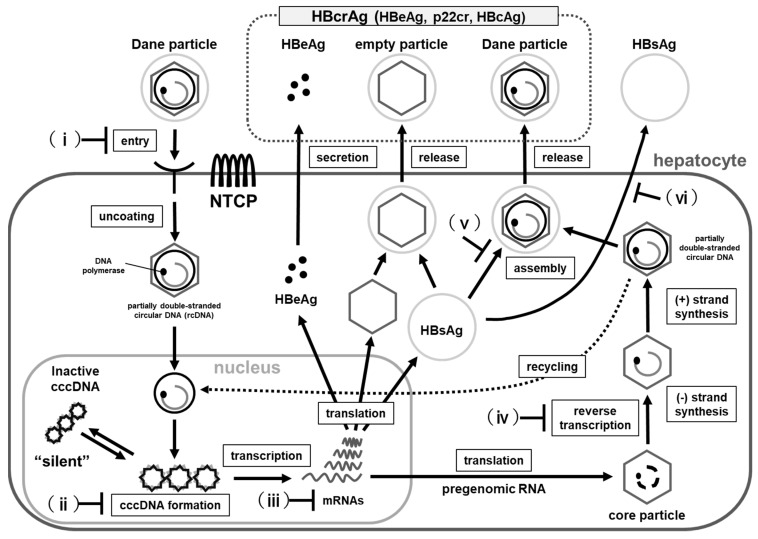
Life cycle of HBV and therapeutic agents. (**i**) Inhibition of HBV entry into hepatocytes, (**ii**) Targeting cccDNA, (**iii**) Targeting viral transcripts, (**iv**) Inhibition of reverse transcription, (**v**) Inhibition of capsid formation or nucleocapsid assembly, (**vi**) HBsAg Inhibitors.

HBV enters into hepatocytes, mediated by the attachment of the pre-S1 region of the surface protein to the hepatocellular sodium taurocholate co-transporting polypeptide (NTCP) (Figure 1) [8]. Next, the virion is uncoated and transferred into the nucleus of the hepatocyte. The HBV genome, which was relaxed circular DNA (rcDNA) or linear DNA in the virion, is transformed into cccDNA through covalent ligation in the cell nucleus [9]. cccDNA contributes to viral persistent infection and is the most important factor preventing viral elimination by therapy. The viral mRNAs and pregenomic RNA are transcribed using cccDNA as the template. The mRNAs are translated into viral proteins and the pregenomic RNA is reverse transcribed into HBV genomic DNA [10]. cccDNA copies are replicated via the reverse transcription pathway in the cytoplasm [11]. The mature nucleocapsids are transferred to the nucleus and recycled or secreted as Dane particles [10,12,13,14].

Chronic HBV infection consists of five stages [15]. Stage 1 is an immune tolerant stage and, generally, treatment is not indicated. In this stage, HBV DNA, HBsAg, and HBeAg are detectable in the serum [16]. Only an antibody to hepatitis B core antigen (anti-HBc) is produced [17]. The serum alanine aminotransferase (ALT) level is rarely increased.

Stage 2 is an immune active/clearance stage. Serum ALT levels are high, hepatic necrotizing inflammation occurs, and fibrosis progresses rapidly, so treatment should be considered [4,15,16,18,19]. HBeAg can be detected in the serum [16]. The immune response suppresses HBV replication and begins to reduce HBsAg and HBeAg. HBeAg clearance (seroconversion to anti-HBe) occurs in 10–20% per year. Stage 2 ends with HBeAg seroconversion [15].

Stage 3 is an inactive chronic infection stage. In this stage, HBV DNA is often undetectable in the serum and ALT is not elevated [15], but HBsAg is detectable in the serum. Clearance of HBsAg can occur naturally in 1–3% of cases per year [20].

Stage 4 is an immune escape stage. Even though HBeAg is equally undetectable, HBe-negative chronic hepatitis has a different clinical course from inactive HBV carriers because of the replication of HBV variants that do not produce HBeAg, and the risk of developing decompensated cirrhosis and HCC is not low [15].

Stage 5 is a reactivation or acute–on–chronic hepatitis stage. Reactivation of HBV can be caused by systemic chemotherapy or immunosuppressive therapy. Patients who have cleared HBsAg and have undetectable HBV DNA but are positive for anti-HBc may have HBV reactivation when they are treated with potent immunosuppressive therapies (de novo hepatitis B) [21].

As described above, HBV cannot easily be removed from the liver because of the persistence of cccDNA [22]. The amount and transcriptional activity of cccDNA in hepatocytes are closely related to pathogenesis and progression [23]. Therefore, the evaluation of serum HBV biomarkers related to cccDNA is of clinical value [23].

## 3. Current and New Therapies for CHB

Although therapies, such as IFNs and NAs, have been developed, no treatment has been able to eliminate HBV from the host cells. Therefore, it is necessary to regularly monitor the response during and after treatment [24]. In general, the therapeutic approaches to limit HBV replication can be categorized as drugs targeting the virus directly (direct-acting antivirals) or indirectly via modulation of the host immune response (immunotherapy). In this section, we will focus on current treatments for CHB and a few potential new therapeutic agents, particularly as they relate to their effects on the intrahepatic replication cycle of HBV (Table 1).

Therapeutic agents for HBV in the liver are roughly as follows in Figure 1: (i) Entry inhibitors, (ii) Targeting cccDNA, (iii) Targeting viral transcripts, (iv) Targeting HBsAg, (v) Targeting viral nucleocapsid assembly, and (vi) HBV polymerase inhibitors. In particular, capsid assembly modifiers (CAMs) are promising drugs because they not only directly inhibit core proteins in the liver but may also inhibit de novo synthesis of cccDNA.

### 3.1. Current Treatments for CHB

Current treatments for CHB include injectable IFN (PEG-IFNα2a) and NAs (entecavir and tenofovir), which are oral and direct-acting antiviral agents to HBV.

Interferon is a treatment for young patients with preserved liver function who do not want long-term treatment. The advantages of interferon compared to NAs are the limited duration of treatment, once a week for 48 weeks, the lack of selection of resistant mutants, and the higher rate of HBeAg and HBsAg clearance induction than NAs [25]. Therefore, HBsAg and HBeAg are mainly monitored for their effects and their disappearance is one of the goals of treatment. On the other hand, the adverse events of interferon can be severe in many cases, making it difficult to administer to patients with decompensated cirrhosis or portal hypertension.

Several types of NAs are currently available (Table 1). The main advantages of entecavir are its potent antiviral activity and low drug resistance in newly treated cases (about 1% after 5 years of treatment). On the other hand, when entecavir is given to lamivudine-resistant patients, resistance has been observed in up to 50% after 5 years of treatment. Tenofovir is available in two formulations, tenofovir disoproxil (TDF) and tenofovir alafenamide (TAF). Although there is more evidence regarding TDF compared to TAF, TAF is as effective as TDF while having less renal and bone toxicity than TDF [26,27,28]. Tenofovir is less likely to induce resistance mutations and can be used as first-line therapy in treatment-naïve or lamivudine-resistant patients. Although lamivudine and adefovir are less expensive than other oral agents, they are not currently used preferentially due to drug resistance and nephrotoxicity.

Since NA treatment decreases HBV-DNA but not HBsAg, its therapeutic goal is to make serum HBV-DNA unmeasurable. On the other hand, HBcrAg, which is unaffected by NA, may be useful for monitoring the activity of HBV-cccDNA in hepatocytes during NA treatment.

### 3.2. Core Protein and Capsid Assembly Modulators

The HBV core protein, which has multiple roles in the viral replication cycle, is one of the most promising targets in the current development of anti-HBV drugs [29]. Several compounds which are currently under development, called core protein and capsid assembly modulators (CAMs), bind to the hydrophobic pockets of the capsids and inhibit nucleocapsid assembly, pregenomic RNA encapsidation, or both. As a result, synthesis of rcDNA from pgRNA is inhibited [30,31].

Class I CAMs induce the formation of capsids that are assembled incorrectly. Class II forms empty capsids that are morphologically normal but lack pgRNA and HBV polymerase. Currently, a variety of CAMs are under development (Table 1).

RO7049389, a class I CAM, induces the formation of aberrant HBV core aggregates and causes defects in capsid assembly, thereby inhibiting HBV replication. NVR 3-778, a sulfamoylbenzamide derivative, was shown to reduce HBV DNAs and RNAs in HBeAg-positive CHB patients, however, a rebound in viral activity was observed after treatment was discontinued [32]. JNJ-6379 was effective in treatment-naïve CHB patients without cirrhosis. ABI-H0731, an oral core protein inhibitor, caused a significant reduction in the levels of HBV RNA in HBeAg-positive patients receiving NA. BAY41-4109 showed antiviral activity against various panels of clinical isolates of HBV genotypes A-H, and the effect of amino acid substitutions in the core protein was evaluated using site-specific mutants [33]. These results suggest that CAMs constitute a potential new class of therapeutic agents that act on the transcriptional activity of liver core proteins and possibly cccDNA.

### 3.3. RNA Interference (RNAi)-Based Therapy and Antisense Molecules

Prolonged exposure to high viral antigen levels may exhaust the host immune response, leading to the persistence of HBV infection. Therefore, the therapeutic approaches targeting viral RNA may be an effective strategy to control HBV infection [34].

Because all transcripts delivered from cccDNA share a common 3′ end, targeting this region with RNA interference can target all HBV mRNAs. Several therapeutic agents using siRNAs have been designed (Table 1). A dynamic polyconjugation (DPC) platform, developed to deliver therapeutic RNAi trigger molecules, was used for ARC-520 and ARC-521 [35,36,37]. The siRNAs were designed to reduce antigen production and allow a potential host immune response and functional cure [38,39]. Trials of ARC-520 considered that interfering with viral transcripts reduces antigen production in CHB patients [40]. ARC-520 also revealed vicariously that integrated viral DNA in the host genome can be a source of HBsAg [38]. Furthermore, clinical trials of combination treatment with ARC-521 and entecavir showed good tolerability and reduction in HBV DNA, HBsAg, and cccDNA in CHB patients [41]. However, trials using this compound stopped due to the potentially lethal toxicity of the delivery vehicle in nonhuman primates.

Another approach to blocking the expression of the viral proteins is liver-directed antisense oligonucleotides, aimed at RNA degradation. The antisense oligonucleotides, GSK33389404 and GSK3228836, bind to a N-acetylgalactosamine (GalNAc) and are delivered into the liver via asialoglycoproteins expressed by hepatocytes. Such an approach may reduce the off-target toxicity [42]. RG7834 is a novel oral inhibitor of HBV viral gene expression, belonging to the dihydroquinolizinone chemical class, and has been shown to inhibit PAPD5/7 in HBV infected human liver chimeric uPA/SCID (PXB) mice. The agent causes highly selective inhibition of HBV transcription in this mouse model [43].

Neither siRNA nor antisense oligonucleotides eliminate cccDNA and a rebound to pretreatment levels of HBsAg has been observed after treatment cessation. Therefore, attention to the persistence of response and combination therapy with agents using other mechanisms will be required [44].

### 3.4. CRISPR-Cas9-Related Therapy

In the nucleus, viral cccDNA has chromatin-like structures and is epigenetically regulated to serve as the template for viral transcription [45]. Strategies to inhibit or destroy cccDNA formation and silence transcription are being investigated. However, it is difficult to eliminate HBV because of the high stability of cccDNA.

CRISPR-Cas9 and other genome editing technologies have contributed to basic genomic and clinical research, such as genetic recombination and viral inactivation [46]. Highly multiplexed CRISPR-Cas9-nuclease and Cas9-nickase have been developed to target three major domains of the HBV genome simultaneously [47]. Transfection of the all-in-one vectors caused fragmentation of the HBV genome and significantly reduced HBeAg and HBsAg levels [48]. Despite its high efficacy in inhibiting the replication of HBV, off-target mutations in the host genome were not detected by genome truncation detection assays, and a small number of mutations were detected only by deep sequencing analysis. Therefore, the all-in-one vectors provide a model that simultaneously targets multiple HBV domains and may contribute to an appropriately designed therapeutic approach to treat HBV patients [46].

On the other hand, several compounds unrelated to genome editing have been shown to repress cccDNA transcription in vitro. Interferon-α inhibits the transcription of genomic RNAs from cccDNA in HBV infected hepatocytes derived from PXB mice [49]. An essential part of viral transcription is mediated by the degradation of the host structural maintenance of the chromosomal (Smc) complex 5/6, which selectively blocks extrachromosomal DNA transcription and gene expression [50,51]. Indeed, the use of pevonedistat, an NEDD8 activating enzyme inhibitor that restores levels of Smc5/6 protein, suppressed viral transcription in hepatocytes in vitro [52].

Furthermore, induction of APOBEC3A/B by cytokine stimulation, such as by interferon-α and phosphotoxin-b, has been shown to cause a decrease in cccDNA in vitro [53,54]. In addition, targeting HBX may be also useful to silence cccDNA.

The therapeutic strategy of targeting cccDNA has been shown to be effective in in vitro studies. Specificity for viral targeting and efficient and safe delivery of gene editing agents to remove cccDNA from all infected hepatocytes are so far incompatible and have not been applied to clinical trials at present [47,55,56,57,58,59,60,61]. Recently, reprogrammable site-specific nucleases, such as transcription activator-like effector with zinc finger domain [62,63], have been reported. The studies based on genome editing technologies are of great interest and have the potential to eliminate cccDNA or to inactivate the transcription from cccDNA, but their efficiency, delivery, adaptability, and specificity need to be improved.

## 4. The Clinical Use of HBcrAg

Testing for HBcrAg has been recommended in clinical guidelines for CHB management in several countries, first in Japan, then in Asia, and recently, in Europe [64,65,66]. Here, we introduce the characteristics of HBcrAg, as well as the configuration of HBcrAg, the association between serum HBcrAg and other HBV biomarkers, especially between HBcrAg and intrahepatic cccDNA, and a high-sensitivity HBcrAg assay which will soon be in clinical use in Japan.

### 4.1. Configuration of HBcrAg

HBcrAg contains three proteins including HBeAg, HBcAg that forms the nucleocapsid in the Dane particle, and an empty particle without DNA (p22cr) [67,68,69,70]. All three proteins encoded by the precore/core region are derived from the same 149 amino acid sequence [69,71]. By serological laboratory testing, HBeAg, an empty particle (p22cr) [72,73], and HBcAg can all be detected as HBcrAg [68,74].

### 4.2. The Relationship between Serum HBcrAg and Other HBV Markers

HBcrAg and other HBV markers are shown in Table 2. Firstly, Kimura et al. showed that the amount of HBcrAg depends on serum HBV DNA levels [67]. Since then, the use of serum HBcrAg monitoring of CHB patients has been proposed by several reports, which show that serum HBcrAg levels are related to HBV DNA levels [68,75,76,77]. That is, the serum HBcrAg concentration precisely indicates the serum HBV DNA levels, irrespective of HBeAg status. The total amount of intrahepatic HBV DNA is also reflected by the serum HBcrAg levels in patients with and without NA treatment [75,77]. In addition, serum HBcrAg levels were strongly correlated with serum HBsAg, HBsAg-HQ, and HBV DNA levels [78]. These findings suggest that the serum HBcrAg level correlates not only with conventional HBsAg assays but with a sensitive HBsAg assay (HBsAg-HQ). Loggi et al. compared the ability of HBcrAg levels with serum HBsAg levels to determine the clinical profile of HBeAg-negative CHB patients [79]; HBcrAg levels in patients with undetectable HBeAg were significantly higher in the CHB patients than the clinically inactive carriers. A cutoff value of 2.5 log U/mL provided a diagnostic precision comparable to serum levels of HBsAg and identified clinically inactive carriers.

Testoni et al. reported that patients with negative HBcrAg had lower amounts and activity of intrahepatic cccDNA than those positive for HBcrAg. In addition, serum HBcrAg levels correlated with serum and intrahepatic HBV DNA, cccDNA, and pgRNA levels, and transcriptional activity, which were significantly higher in HBeAg-positive patients with than in HBeAg-negative patients. Higher HBcrAg levels were correlated with these viral markers, as well as fibrosis and necroinflammatory activity scores [65]. Moreover, even in HBeAg-negative CHB patients, higher levels of HBcrAg were significantly associated with inflammatory activity and fibrosis [80].

### 4.3. HBcrAg as a Marker Which Reflects Intrahepatic cccDNA and Its Transcriptional Activity

No standardized procedure has been developed for the direct evaluation of cccDNA. Therefore, a method to precisely evaluate the amount of intrahepatic cccDNA, using a consistent noninvasive technique, will contribute to a variety of clinical applications. On this basis, serum HBcrAg is thought to be a suitable tool for application in clinical practice to improve the management of patients. Several reports have shown that serum levels of HBcrAg are closely correlated with cccDNA levels, as well as serum levels of HBV DNA [70,75,81,82,83]. Serum HBV DNA levels are correlated with intrahepatic cccDNA levels [77]. However, 78% of the CHB patients treated with NA still remained positive for serum HBcrAg, although they showed undetectable serum HBV DNA [77]. Consequently, on the background of undetectable serum HBV DNA, HBcrAg can be the ideal serum biomarker to assess the amount of intrahepatic cccDNA.

### 4.4. The Relationship between HBcrAg and HBV RNA

Serum HBV RNA usually becomes undetectable before HBcrAg does [84]. Liao et al. assessed the clinical impact of serum levels of HBV RNA and HBcrAg in CHB patients in whom HBV DNA was undetectable with NA treatment. In the study, the levels of HBV RNA were significantly associated with those of HBcrAg but not HBsAg. The samples positive for HBeAg had higher levels of HBV RNA, HBcrAg, and HBsAg than the samples negative for HBeAg (all *p* < 0.05) [84].

In a previous report, the HBV RNA mirrored cccDNA levels in CHB patients positive for HBeAg, and total serum HBV DNA plus HBV RNA reflected better the levels of cccDNA than did serum HBV DNA levels [85]. On the other hand, serum HBcrAg levels correlated with levels of cccDNA better than HBsAg and HBV RNA levels, irrespective of the HBeAg status, although serum levels of HBsAg and HBV RNA differ significantly between CHB patients with detectable HBeAg and those in whom it is undetectable [86]. In another report, Carey et al. also examined the ability of serum levels of HBcrAg and HBV RNA to be surrogate markers for the silencing of cccDNA, to characterize virological outcomes [87]. Their results indicated that serum HBV RNA and HBcrAg are highly sensitive markers of transcriptional activity of cccDNA in HBeAg-negative patients, even when HBV DNA is suppressed under NA treatment [87].

### 4.5. High-Sensitivity HBcrAg Assay

Recently, we developed a novel high-sensitivity HBcrAg assay (iTACT-HBcrAg), a fully automated high-sensitivity CLEIA, to improve the sensitivity of HBcrAg detection and reported that it is useful for early detection of HBV reactivation as well as for monitoring CHB patients with undetectable HBeAg [88]. HBV DNA assays are useful, with high sensitivity and specificity but are expensive and require a long time to produce results [89]. iTACT-HBcrAg is less expensive than HBV DNA assays and easier to use and can evaluate serum HBcrAg levels within 30 min. Furthermore, the sensitivity of iTACT-HBcrAg is approximately 10 times greater than the conventional HBcrAg assay [88].

In another study of iTACT-HBcrAg, Suzuki et al. reported that the proportion of HBcrAg ≥ 2.7 was significantly higher in the HCC group than the non-HCC group, indicating that the remaining low HBcrAg might predict HCC development, even if HBcrAg seroclearance was achieved according to the conventional assay [90].

In cases that show undetectable serum HBV DNA, serum HBcrAg levels are the ideal serum biomarker to assess the amount of intrahepatic cccDNA. We expect that iTACT-HBcrAg will soon be used as a marker in clinics and will be an excellent tool that can more accurately reflect the amount and transcriptional activity of cccDNA.

### 4.6. Prediction of HBsAg Seroconversion by HBcrAg

The recent clinical use of HBcrAg is shown in Table 3. Most patients (79%) with HBsAg seroclearance had undetectable levels of HBcrAg, suggesting a more quiescent state of HBV replication. On the other hand, among the 21% of patients whose serum HBcrAg was still detectable, the median level of HBcrAg was 2.7 log U/mL [78,91]. Although the optimal cutoff has not been determined yet, these findings suggest that HBcrAg may be used to further define the stage of CHB [40].

### 4.7. Monitoring the Effect of Anti-HBV Treatment Using HBcrAg

The development of NAs has made it possible to reduce hepatitis activity and suppress serum HBV DNA [103]. However, no useful biomarkers have been identified to assess the appropriateness of discontinuing NAs and the risk of developing NA resistance in CHB patients. In HBeAg-positive CHB patients, loss of HBeAg and seroconversion to anti-HBe are endpoints worth considering. After positive anti-HBe results, achieving HBcrAg and HBsAg reduction and subsequent HBsAg loss is considered the primary goal of CHB treatment [64].

In HBeAg-negative CHB patients, the goals of antiviral therapy are sustained HBsAg clearance without treatment [92]. However, because it is difficult to achieve HBsAg clearance during NA therapy, a decrease in HBsAg and HBcrAg may be helpful in determining the efficacy of NA therapy.

### 4.8. Assesment of the NA Cessation Point According to HBcrAg

Most NA medicated patients will continue to receive treatment; however, some can choose to cease therapy. The decision to stop NA treatment has traditionally been based on the levels of serological biomarkers, HBV DNA, ALT, and HBsAg. In CHB patients with NA treatment, a decrease in HBcrAg may provide potent information on the risk of HBV reactivation after treatment [104].

A higher HBcrAg level than 3.7 log U/mL at the time of NA discontinuation predicted virological relapse within one year [105]. A similar report describes the results of CHB patients using LAM, where a high HBcrAg level at the time of NA cessation predicted virological relapse, even if serum HBV DNA was undetectable within 6 months [95]. Therefore, serum HBcrAg can provide a better decision-making tool for patients planning to discontinue NAs. Conversely, stopping LAM at the HBcrAg level <3.4 log U/mL significantly predicted the absence of relapse. Furthermore, no patient with HBcrAg <3.0 log U/mL at the time of discontinuation LAM had an ALT flare [85]. The HBcrAg level at the time of discontinuation of ETV is also correlated with relapse [106]. Recently, Sonneveld et al. reported the relationship between end-of-NA treatment levels of HBcrAg and HBsAg and outcome after NA treatment cessation [107]. They concluded that lower levels of HBcrAg and HBsAg were correlated with favorable outcomes, including sustained virological responses [107].

### 4.9. HBV Reactivation by High-Risk Immunosuppressive Therapy

Immunosuppressive treatment is a very important factor in HBV reactivation. It was reported that the risks of HBV reactivation have been stratified [108]: high risk was defined as ≥10%, moderate risk as 1–10%, and low risk as <1%. High-risk regimens involve systemic chemotherapy containing rituximab [109,110,111] and hematopoietic stem cell transplantation (HSCT) [112]. Allogeneic hematopoietic stem cell transplantation (allo-HSCT) is an independent risk for HBV reactivation in patients suffering from hematologic malignancies [113]. In 2001, the first case of lethal HBV reactivation was described in a patient treated with R-CHOP, including rituximab [110]. Notably, patients with non-Hodgkin lymphoma administered very high-risk immunosuppressive therapies are susceptible to HBV reactivation and secondary adverse events [114]. In B-cell depletive therapies, including rituximab, the risk of HBV reactivation is lengthened [115,116]. Moreover, HBV reactivation after HSCT may come about several years after transplantation [112]. Therefore, HBV reactivation may continue over an extended period after immunosuppressive therapy is given, requiring long-term observation. Large-scale prospective studies of HBV reactivation in malignant lymphoma patients administered rituximab containing regimens for have been reported from Japan [109], Hong Kong [116], and Taiwan [117]. Preemptive antiviral therapy, based on HBV DNA monitoring, can prevent hepatitis due to HBV reactivation in patients with resolved HBV infection who receive systemic chemotherapy. Recently, we reported for the first time that iTACT-HBcrAg is useful for the early diagnosis of HBV reactivation [88].

### 4.10. HBcrAg as a Predictor of HCC Occurrence and Recurrence

It is not easy to predict which patients on NA treatment will develop liver-related events, including liver carcinogenesis [103]. Higher titers of HBV DNA have been associated with a higher risk of HCC development [118]. On the other hand, if the HBV viral load is low or undetectable, the risk of developing HCC is reduced but not completely prevented [100,119]. According to a large cohort study, a high level of HBcrAg predicted the development of HCC in treatment naïve CHB patients more accurately than did HBV DNA [97]. During the period of followup (10.7 years), HCC occurred in the 7.6% of CHB patients without NA treatment. HBcrAg above 2.9 log U/mL was independently correlated with HCC occurrence [97]. HBcrAg 4.0 log U/mL was also reported in another study as an independent risk factor of HCC in patients with intermediate HBV DNA levels (from 2000 to 19,999 IU/mL) [120]. In CHB patients treated with NA for at least two years, HBcrAg was an independent risk factor for development of HCC [99]. In patients with undetectable HBV DNA treated with NAs, HBcrAg levels were significantly higher at baseline in the HCC group than the coordinated control group. Higher levels of HBcrAg post-treatment also predicted HCC occurrence [98]. A study examining the long-term effect of NA therapy on the progression of HCC in CHB patients showed that higher HBcrAg levels were significantly correlated with HCC progression, independent of NA therapy [121]. There are few reports about the relationship between HCC recurrence and HBcrAg. The rates of postsurgical HCC recurrence remained high despite NA treatment, with reported recurrence rates of up to 41.8% over more than two years [122]. It was also reported that HCC patients with high intrahepatic cccDNA and serum HBcrAg levels had a significantly lower HCC recurrence-free survival rate than those with low cccDNA/HBcrAg levels [123].

## 5. Discussion and Prospects

### 5.1. HBcrAg Silencing as a Therapeutic Goal for HBV

For prevention of carcinogenesis, one of the therapeutic goals for HBV is to reduce HBV DNA and HBsAg levels. The sequential goals of HBV treatment are first HBV DNA negativity, then HBsAg negativity and, finally, HBV cccDNA elimination. However, it is not easy to eliminate HBV, which has double-stranded DNA, because we are a nucleated organism with double-stranded DNA. The transcriptional activity of cccDNA is regulated by epigenetic mechanisms similar to human DNA, such as DNA methylation and histone modification, and cccDNA has an epigenetically active/inactive state. When cccDNA becomes epigenetically inactive, “silent cccDNA” the transcriptional activity of cccDNA decreases and HBV RNA becomes negative.

It is more realistic to reduce the transcriptional activity of cccDNA or HBV RNA, as a therapeutic target, than to eliminate cccDNA, which is considered difficult at present. In addition, considering the complexity of measuring HBV RNA, it is useful to consider this as HBcrAg negativity. Therefore, lowering HBcrAg reflects the epigenetically inactive state of HBV. In other words, “HBcrAg clearance”, HBcrAg negative by a high-sensitivity assay, indicating a state in which HBV is epigenetic inactive, may be important as a therapeutic goal. As mentioned in this article, when HBcrAg is low, the therapeutic effect is good and hepatocarcinogenesis and HBV reactivation are unlikely to occur. An effective strategy to achieve this is desirable.

### 5.2. Usefulness for Evaluating Combination Therapies in Patients Receiving NA Therapy

Treatment with NAs has made it easy to achieve undetectable HBV DNA. Although the treatment does not eliminate cccDNA, it is very good at suppressing HBV DNA replication and it has few side effects, unlike IFN. Currently, various therapeutic agents are being developed and these may be used in combination with NAs. Therefore, cocktail treatment based on NAs with antiviral agents targeting other steps in the HBV lifecycle will be necessary to achieve a functional cure.

One possible combination for complete inhibition of intrahepatic HBV replication could consist of an NA plus one or two other direct-acting antiviral agents with different mechanisms, such as CAM, siRNA, cccDNA inhibitors, and entry inhibitors. The transcriptional activity of cccDNA should be evaluated in clinical trials. However, it is not sufficient as a marker for developing combination therapy because NA medication masks HBV DNA. In addition, it is not ethically appropriate to delay the opportunity of NA internalization due to clinical trials. On the other hand, HBcrAg reflects the amount and transcriptional activity of cccDNA in hepatocytes and is not affected by NAs, suggesting that it is ideal for monitoring the activity of chronic hepatitis B patients in NA combination therapy trials. This may contribute to the development of important drugs for HBV therapy in the future.

### 5.3. Another Therapeutic Approach, Immunotherapy

This review has mostly focused on therapies targeting the virus directly (direct-acting antivirals); however, immunotherapy, modulation of the host immune response, is another therapeutic approach to treating HBV infection [124]. Relieving T cell exhaustion, caused by sustained exposure to viral antigens in chronic HBV infection, may lead to viral elimination. The strategies to induce immunity include cytokines, chemokines, and pattern recognition receptor agonists that induce the production of interferon and approaches to reconstituting HBV-specific immunity using checkpoint inhibitors and therapeutic vaccines. In addition, combining these immunological approaches with direct-acting antiviral agents that target HBV replication may also lead to a more efficient recovery from exhaustion. In aiming to relieve such exhaustion, HBcrAg clearance, which reflects silencing of hepatic cccDNA replication activity, will be helpful to ensure that the virus is free from intrinsic and persistent stimulation.

## 6. Conclusions

Serum HBcrAg is a useful novel HBV biomarker. In particular, serum HBcrAg is a suitable surrogate biomarker to reflect the amount and replication activity of hepatic cccDNA. Additional trials and studies are desired to investigate the use of the high-sensitivity HBcrAg assay in many aspects of HBV clinical practice. Prospective studies are needed comparing the long-term outcomes of HBcrAg-positive and HBcrAg-negative patients, according to the high-sensitivity assay, especially in patients with undetectable HBV DNA and HBsAg. Furthermore, followup using the high-sensitivity HBcrAg assay is expected to be a very useful prognostic factor for predicting the long-term outcome of CHB.

## Figures and Tables

**Table 1:** HBV therapeutic agents targeting HBV directly (Hepatitis B Foundation: Drug Watch: https://www.hepb.org/treatment-and-management/drug-watch/ (accessed on 7 August 2021).

	Category or Target	Mechanism	Drug Name
(1)	Viral entry	Interferes with HBV getting into liver cells	Bulevirtide (Hepcludex®)
(2)	cccDNA	Intended to destroy or repress HBV-cccDNA	EBT-106, EBT-107
(3)	Viral RNA	Silencing RNA’s (siRNAs)	AB-729, ARC-520, ARC-521, ALG-125097, ALN-HBV, ARB-1467, ARB-1740, BB-103, BB-HB-331, JNJ-3989, Lunar-HBV, RG6346, VIR-2218
		Antisense Molecules	GSK 3228836, ALG-020572
(4)	HBsAg release Inhibitors	Synthetic oligonucleotides that bind HBsAg	ALG-10133, REP 2139, REP 2165
(5)	Core protein and capsid	Assembly modulators	ABI-H0731, ABI-H2158, ABI-H3733, ALG-000184B-836, BAY41-4109, BCM-599, Morphothiadin, EDP-514, GLP-26, JNJ 6379, NVR 3-778, QL-007, RG7907, RO7049389, VNRX-9945, ZM-H1505R,
(6)	Reverse transcription	Nucleos(t)ide Analogues	Lamivudine, Adefovir dipivoxi, Entecavir, Telbivudine, Tenofovir disoproxil, Tenofovir Alafenamide, Cledvudine, Zadaxin

**Table 2:** The Pros and Cons of HBV biomarkers.

Marker	Diagnosis	Pros and Roles	Cons and Disadvantages
HBsAg	Being Infected with HBV	Easy to measure and highly sensitive	Useless against escape mutants
		Useful for diagnosis of infection	Does not always reflect the amount of HBV
			False positives have increased as a result of highly sensitive assays
HBsAb	Having been infected with HBV	Acts as a neutralizing antibody against HBsAg	Unable to distinguish previous infection from vaccination
	After vaccination	Protection against HBV infection	Possible negative in previous infection
HBcAb	Having been infected with HBV	Persistent antibody titer	Not suitable as a marker for acute phase
		Most sensitive marker of infection	Conventional methods may not be sensitive enough.
		Does not become positive after vaccination	
IgM-HBcAb	High titer: acute hepatitis	Useful for diagnosis of acute hepatitis and differentiation of acute on chronic hepatitis	Useless for diagnosis of previous infection
	Low titer: acute exacerbation of chronic hepatitis		
HBeAg	High HBV activity	Indicates that the virus is in a state of active replication	Difficult to predict HBeAg seroconversion in the case with pre-core and core-promoter mutation
HBeAb	Low HBV activity	Indicates that viral replication has slowed and infectivity has decreased	HBe antibodies remain positive even as HBV increases in the case with pre-core and core-promoter mutation
HBcrAg	Reflect the amount and the activity of cccDNA	Easy to measure	Not well known due to insufficient sensitivity (conventional method)
		High sensitivity (iTACT-HBcrAg)	
		Useful for monitoring of HBV transcriptional activity even under NA medication	
HBV-DNA	Reflects the amount of HBV	Useful for monitoring the amount of HBV	Useless for monitoring ofHBV transcriptional activity under NA medication
		High sensitivity	
HBV-RNA	transcriptional activity of HBV	Useful for monitoring of HBV transcriptional activity	Unstable
			Findings are covered by HBcrAg

**Table 3:** Clinical applications of HBcrAg.

Category	Application	HBcrAg Level and Point	References
Natural history	Seroconversion to HBeAg negative	Less than 4.92 log U/mL	[78]
	Seroclearance of HBsAg	Undetectable (79%), and 2.7 log U/mL (21%)	[78,91]
	Seroclearance of HBcrAg	Undetectable HBcrAg	[88]
Anti-HBV treatment	Seroconversion to HBeAg negative (induced by PEG-IFN at 12 weeks)	Larger than 8 log U/mL at the beginning of the therapy	[92]
	Seroconversion to HBeAg negative (induced by PEG-IFN plus NA)	Larger than 4.5 log U/mL at the beginning of the therapy	[93]
	Relapse within a year after NA cessation	Larger than 3.7 log U/mL at NA cessation	[94]
	Relapse in spite of undetectable HBV DNA for at least 6 months	From 3.2 to 3.7 log U/mL at NA cessation	[95,96]
HCC development/diagnosis	Incidence of HCC (treatment-naïve patients)	Larger than 2.9 log U/mL during the follow-up	[97]
	Incidence of HCC (treatment-experienced patients)	Larger than 4.67 log U/mL at pretreatment, and Larger than 3.89 log U/mL at post-treatment	[98]
	HCC development with NA treatment	Detectable HBcrAg during NA treatment	[99]
HCC recurrence	HCC recurrence within 2 years	Larger than 4.8 log U/mL at the time of HCC diagnosis	[100]
HBV reactivation	HBV reactivation by high-risk immunosuppressive therapy within 2 years	Detectable HBcrAg before therapy	[101]
	High levels of cccDNA after liver transplantation	Larger than 4 log U/mL before liver transplantation	[102]
Clinical Trial of new therapy	Evaluation of new therapeutic agents for the patients under NA administration	Undetectable HBcrAg at post-treatment	[88]

Abbreviations: HBcrAg, hepatitis B core-related antigen; HBV, hepatitis B virus; PEG-IFN, pegylated interferon; NA, nucleos(t)ide analogue; LAM, lamivudine; ETV, entecavir; HCC, hepatocellular carcinoma; cccDNA, covalently closed circular DNA.
